# Real-time monitoring of photodegradation in photoresists using a quartz crystal microbalance†

**DOI:** 10.1039/d4ra05762g

**Published:** 2025-04-17

**Authors:** Zhun Gu, Kaitong Yang, Hayford Boamah, Dong Chen, Zhiqiang Zhu, Jie Wang

**Affiliations:** a School of Biomedical Sciences, Suzhou Chien-shiung Institute of Technology 1 Jianxiong Road Suzhou 215411 China zhuzq@csit.edu.cn; b Institute for Advanced Materials, Jiangsu University Zhenjiang 212013 China wangjie@ujs.edu.cn

## Abstract

We report a quantitative method for the real-time evaluation of photoresist performance that integrates laser irradiation with quartz crystal microbalance (QCM) sensing technology. The results obtained for the model photoresist AZ1518 correlated with the traditionally obtained ones. Furthermore, the system revealed viscoelastic transitions and shear stress evolution during photoreactions.

## Abstract 2

By combining a laser and a QCM sensor, this study presents a convenient method for evaluating the photodegradation of the photoresist AZ1518. Moreover, the contrast, shear force and viscoelasticity values for AZ1518 were obtained.

## Graphical abstract

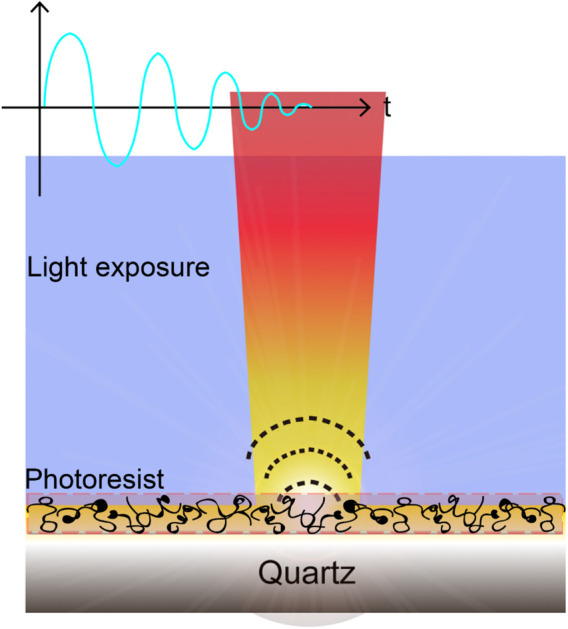

In recent years, the development of micro- and nano-technologies has led to a revolution in the integration and application of various devices.^1,2^ Multifunctional platforms at the nanoscale, such as flexible electronics,^3–5^ solar cells,^6,7^ photonic crystal devices^8–10^ and biomedical electronics,^11–13^ have aroused great interest among researchers. Meanwhile, the sustained evolution of photolithographic technology has fundamentally transformed advanced manufacturing strategies, particularly in establishing critical dimensional control for next-generation semiconductor device architectures.^14^ For the semiconductor industry, photolithography and applied photoresists are fundamental tools and materials. A photoresist, which is usually composed of a polymer, a sensitizer, and a solvent (also called a developer), is used in photolithography to form a patterned coating on the substrate surface.^15^ The lithography process usually starts with spin-coating a photoresist over the substrate surface. Then, a patterned mask is applied onto the substrate surface to shield light. Under UV exposure, photochemical reaction of the positive/negative resists results in the increased or reduced solubility of the developer. The advancement of lithography technology has led to a requirement for higher quality photoresists. Optimizing photoresist contrast is one of the oldest and most commonly adopted strategies to improve the lithographic process. The conventional method for evaluating photoresists is a time-consuming process that involves a number of tedious operations, such as spin-coating, baking photoresist films and measuring the photoresist thickness and reflectivity.^16^ Moreover, methods based on the analysis of the resist thickness *versus* exposure usually require specific conditions.^17^ The difficulty in evaluating resists hinders the development of novel photoresists.

The quartz crystal microbalance (QCM) is a real-time, non-label and highly sensitive sensor. The QCM has been applied in many fields, such as vacuum coating, environmental pollutant monitoring^18^ and biomolecule detection, including protein and DNA detection.^19–25^ Under vacuum or air conditions, according to the Sauerbrey equation, mass change can be calculated from the shift in resonance frequency.^26^ In liquids, the mass change, viscoelastic property and thickness change of analytes can be analysed *via* the Voigt model. Thus, monitoring and analysing the photoreaction of photoresists by the QCM is reasonable because the reaction is accompanied by rapid and vigorous changes in the viscoelastic and shear moduli of photoresists during the photoreaction process.

In this study, we applied a QCM to assess the performance of photoresists in the lithography process. To the best of our knowledge, this is the first application of the QCM platform in this field. We originally combined a laser and a QCM sensor to detect the photochemical process of a photoresist under light irradiation.

A schematic of the real-time online monitoring of the photoresist performance is shown in Fig. 1a. Briefly, the QCM system was placed in a dark box with a laser over the QCM transparent chamber. In the measurement, a series of lasers with wavelengths of 365, 405 and 808 nm was applied (FUV-6BK, Bangwo Technologies Co., Ltd, China) and fixed over quartz resonators. Under periodic UV irradiation, a repeatable frequency shift signal in water was observed (Fig. 1b). This frequency shift was related to the wavelength and intensity of the light exposure. As shown in Fig. 1c, excitation light of a shorter wavelength and stronger power could trigger a stronger frequency shift. After a period of irradiation, the frequency of the QCM tended to stabilize under the light irradiation. The QCM platform could monitor the changes in light, including the wavelength and intensity during the lithography process, providing a good reference for photoresist studies.

**Fig. 1:**
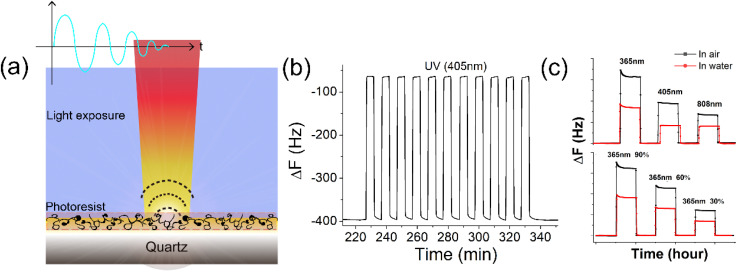
(a) Schematic of the photoacoustic effect of a quartz resonator. (b) Resonant frequency curve under 405 nm irradiation in water. (c) Diagram showing that the shifts in the laser wavelength and exposure intensity lead to frequency shifts.

Typically, to evaluate photoresist performance, Hurter and Driffield proposed the Hurter–Driffield (H–D) curve, a plot of the optical density *versus* log-exposure, to describe the response of a sufficiently thick film of a photosensitive material under light exposure (Fig. 2a). This definition of photoresist contrast (*γ*) was used to assess the ability of a photoresist to distinguish between exposed and unexposed regions under exposure, as determined using eqn (1).1
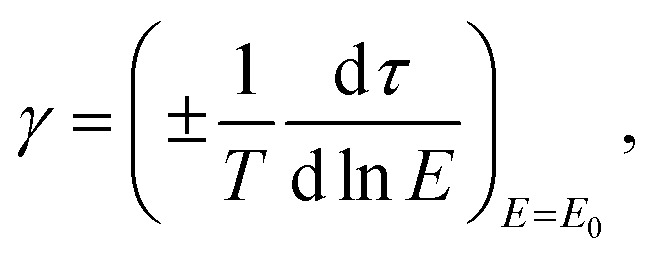
where *T* is the photoresist thickness before development, *τ* is the remaining thickness after development, *E* stands for the nominal exposure energy and *E*_0_ is the energy when *τ* reaches zero. The positive sign is used for negative photoresists, and the minus sign is used for positive photoresists.^27^ This divergence originates from their distinct photochemical pathways: negative photoresists undergo radiation-induced crosslinking, augmenting the film density; while positive photoresists experience chain scission, reducing the density.

**Fig. 2:**
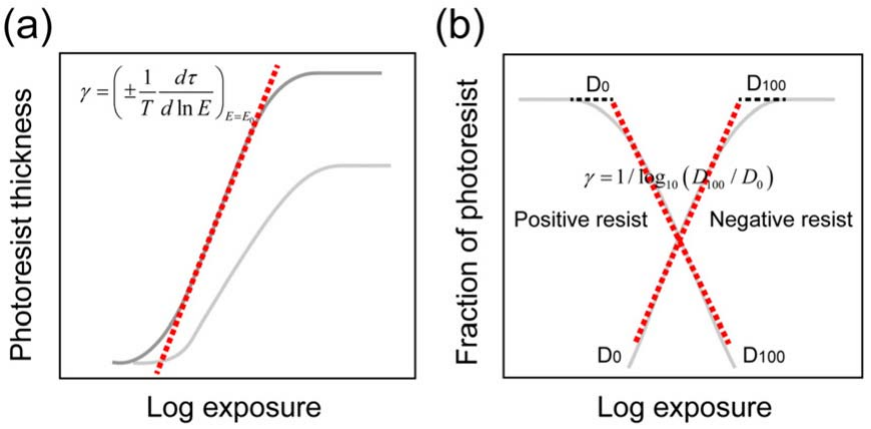
(a) H–D curve proposed by Hurter and Driffield showing the optical density of two negative photographic plates as a function of log exposure. (b) Contrast curve plot of the photoresist thickness *versus* exposure energy.

Photoresist contrast is a simple parameter of how well the irradiated photoresist can perform sharp-edged lithography. Clearly, a high-contrast photoresist could increase lithography resolution. Factors such as the absorption spectrum, bleaching behaviour and surface reflection should be considered. A photoresist with a high contrast allows a quick transit from the exposed region of the photoresist to the unexposed region. As shown in Fig. 2b, the contrast curve could be transformed to describe the relationship between the remaining amount of a uniformly illuminated photoresist and the logarithm of the exposure dose (eqn (2)):^28^2
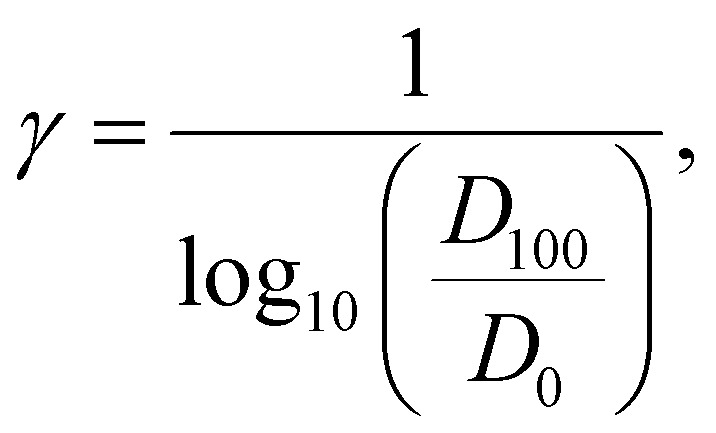
where *D*_0_ refers to the minimum light energy density required for the photochemical reaction and *D*_100_ refers to the minimum light energy density at which the reaction of the photoresist was completed.

Sensitivity is another important parameter used to evaluate photoresist performance. The sensitivity of a photoresist refers to the minimum energy (or exposure dose) of a certain wavelength of light required for producing a pattern on the photoresist. The sensitivity of a photoresist is particularly important for short-wavelength deep ultraviolet (DUV) and extreme deep ultraviolet (EUV) light. *D*_0_ can indicate the sensitivity of a positive photoresist. A lower *D*_0_ demonstrates a higher sensitivity of the measured photoresist. Contrast here refers to the steepness of the photoresist under UV exposure, as defined in eqn (2).

In our study, the positive photoresist AZ1518 was tested in a liquid state without a soft bake of the photoresist to evaluate its typical photoresist properties. Here, photoresist AZ1518 (500 μL) was injected into the QCM reaction chamber, followed by exposure to 365 nm ultraviolet light. Photochemical reactions were initiated when the photoresist gained enough exposure energy. Specifically, in the case of positive photoresists, this energy surplus triggers a decomposition process, wherein the photoresist's molecular structure undergoes fragmentation. This process is accompanied by a density reduction of the photoresist film on the QCM chip. For the changes in viscoelastic properties, the decomposition of positive photoresists leads to an unstructured and unconsolidated product. Conversely, for negative photoresists, the same exposure energy prompts a photocrosslinking reaction, fostering the formation of robust interconnections within the material's molecular architecture. The signals of the resonant frequency shift (Δ*F*) and energy dissipation change (Δ*D*) were recorded and used to measure the photoresist contrast and sensitivity during the photolithography process, as shown in Fig. 3.

**Fig. 3:**
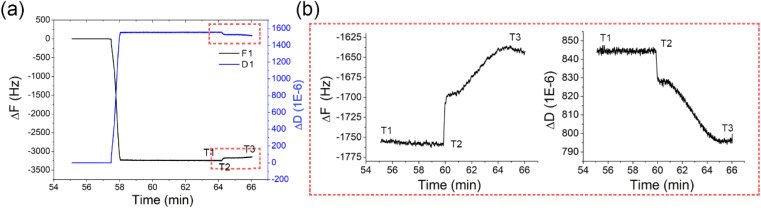
(a) Real-time dynamic monitoring of the UV-induced photochemical reaction of AZ1518 on a QCM chip. (b) Plots of the frequency shift and energy dissipation of the photoresist under 405 nm irradiation.

Fig. 3a shows a plot for the real-time dynamic monitoring of the UV-induced photochemical reaction of AZ1518 on the QCM chip, where *T*_1_ represents the start of the exposure process; *T*_2_ represents the start of the photoreaction, *i.e.*, a decomposition process for AZ1518; and *T*_3_ represents the complete photoreaction. After *T*_3_, the QCM frequency went into a flat state, indicating that the AZ1518 photoresist was completely decomposed within the exposure time (*T*_3_–*T*_1_). The exposure periods *T*_2_–*T*_1_ and *T*_3_–*T*_1_ were used to replace *D*_0_ and *D*_100_ in eqn (2) and (3). The exposure dosage was then calculated by multiplying the light intensity by the exposure time. The smaller *T*_2_–*T*_1_ indicated the higher sensitivity of the photoresist. According to eqn (3), the contrast of AZ1518 could be calculated from *T*_1_, *T*_2_ and *T*_3_.

According to eqn (2) and the physical significance of *T*_1_, *T*_2_ and *T*_3_ in a photolithography experiment, the photoresist contrast parameter can be calculated *via*eqn (3):3
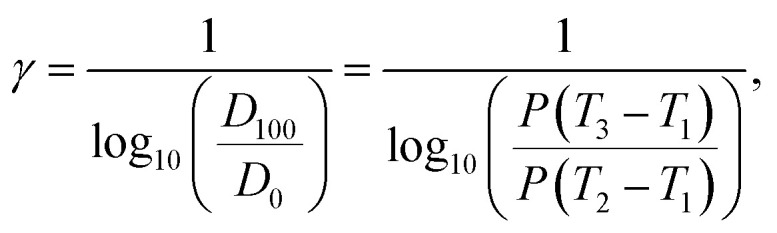
where *P* denotes the light intensity per unit area and unit time, *T*_1_ represents the start of the exposure process, *T*_2_ represents the start of the photoreaction and *T*_3_ denotes the end of the photoreaction of the photoresist.

Fig. 3b shows the real-time frequency and dissipation changes caused by the degradation of the photoresist AZ1518. At first, the AZ1518 liquid flowed through the QCM chip surface, formed a film and induced a frequency shift of about 1700 Hz at the third overtone (*F*_3_). Next, this photoresist film was exposed to UV light at *T*_1_, and it started to decompose at *T*_2_, with a duration (*T*_2_–*T*_1_) of 279 s. The photosensitive constituents of the photoresist underwent photon-induced activation during this phase, initiating scission of polymer backbones. Following time point *T*_2_, a rapid increase in the frequency curve was observed, suggesting continuous decomposition of the photoresist, which resulted in a progressive reduction in the density of the QCM chip's film. Thereafter, the frequency curve gradually stabilized at *T*_3_, with a duration (*T*_3_–*T*_1_) of 572.4 s. Δ*D* was about 5.2 × 10^−6^, which reflected that the structured and rigid photoresist film was broken and a sparse film with a smaller density formed.

According to eqn (3), the exposure dosages, designated *D*_0_ and *D*_100_, can be individually derived through the multiplication of the light intensity quantified in milliwatts per square centimetre (mW cm^−2^) by their corresponding exposure duration. Specifically, *D*_0_ was computed by multiplying the light intensity by the temporal span from *T*_1_ to *T*_2_ (*i.e.*, *T*_2_–*T*_1_), while *D*_100_ was similarly calculated using the duration extending from *T*_1_ to *T*_3_ (*i.e.*, *T*_3_–*T*_1_), with both durations expressed in seconds (S). In our system, a laser with a light intensity of 100 mW cm^−2^ was employed. The QCM could only detect the signals of a liquid layer of about 100 nm on the QCM chip. However, an approximately 1 mm film of photoresist existed on the QCM chip, which would attenuate UV irradiation along the incident direction of light during exposure. To accurately calculate the sensitivity (*D*_0_), a specially designed QCM chamber with an internal cavity height of 1–4 μm should be used. Herein, we discuss the feasibility of QCM analysis for determining the photoresist contrast parameter according to eqn (3):*D*_0_ = *P* × 279 mJ cm^−2^*D*_100_ = *P* × 572.4 mJ cm^−2^Photoresist contrast = 3.21.

Our analysis results are very close to typical values for photoresists.^29^ Notably, considering QCM analysis for photoresists in the liquid state is a novel method, comparing different contrast values of a photoresist *via* different detection techniques may not be beneficial.

In addition, viscoelasticity and shear modulus are important mechanical properties of photoresists and are heavily influenced by the temperature, exposure dose and chemical components of the photoresists. As shown in Fig. 4, the shear modulus and viscoelasticity of the photoresists were simulated *via* a Voigt model using the QCM frequency and energy dissipation data at multiple overtones (1, 3, 5, 7, 9, and 11 overtones). As shown in Fig. 4a and b, cross-harmonic analysis revealed the overtone-independent concordance of frequency perturbations (Δ*f*_n_/*n*, *n* = 3, 5, 7, 9, 11, and 13) and the tendency of dissipative shifts, quantitatively verifying homogeneous photoresist thinning. In Fig. 4c, the decline in the viscoelasticity curve agreed well with the frequency curve, indicating the photoresist had decomposed. The spike of the shear force curve suggested that a shear-force change occurred due to molecular fragmentation of the photoresist. These multiparametric signatures conclusively evidence the scission of the polymer backbone.

**Fig. 4:**
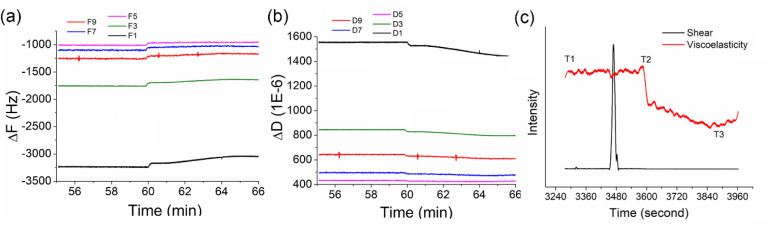
(a) Resonant frequency and (b) energy dissipation of AZ1518 under 405 nm irradiation. (c) Shear and viscoelasticity of the photoresist AZ1518.

This study aimed to provide an easy-to-handle and cost-efficient method not only for assessing the optical contrast of photoresists, but also for analyzing the dynamic photochemical reaction process. Unlike traditional methods, this QCM-based method does not require extra procedures, such as photoresist spin-coating or soft baking before measurement or monitoring of the photoresist film thickness and reflectivity, which are inevitable in other conventional measurements.

## Data availability

Data supporting the findings of this study are available from the corresponding author upon reasonable request.

## Conflicts of interest

There are no conflicts to declare.

## Supplementary Material

RA-015-D4RA05762G-s001
