# Supplementary material for: Real-time monitoring of photodegradation in photoresists using a quartz crystal microbalance
Source: RSC Adv. 2025 Apr 17;15(16):12304–8. doi: 10.1039/d4ra05762g (PMC12004366; doi:10.1039/d4ra05762g)
Supplement: RA-015-D4RA05762G-s001 [file RA-015-D4RA05762G-s001.pdf]

Supplementary Information (SI) for RSC Advances.
This journal is © The Royal Society of Chemistry 2025

Electronic Supplementary Information

Real-Time Monitoring of Photodegradation in Photoresist Using a Quartz Crystal Microbalance

Zhun Gu,[a] Kaitong Yang,[b] Hayford Boamah,[b] Dong Chen,[b] Zhiqiang Zhu*[a] and Jie Wang*[b]

[a] School of Biomedical Sciences, Suzhou Chien-shiung Institute of Technology, 1 Jianxiong Road, Suzhou 215411, China. E-mail: zhuzq@csit.edu.cn

[b] Institute for Advanced Materials, Jiangsu University, Zhenjiang, 212013, China. E-mail: wangjie@ujs.edu.cn

1. Methods

1.1. Materials

All of the Quartz crystal microbalance (QCM) measurements were accomplished on a Q-Sense E4 (Biolin Scientific, Sweden) with a series of lasers. In the measurement, a serial of lasers including 365 nm, 405 nm and 808 nm lasers (FUV-6BK, Bangwo Technologies Co.,LTD, China) were connected with the quartz resonators. The photoresist solutions were controlled by a peristaltic pump set at a speed of 50 μL/min. The quartz resonator with a doping layer of 50nm-thickness Au has a resonant frequency of 5 MHz. The photoresist AZ1518 (Merck, Darmstadt, Germany) was provided by Suzhou Institute of Nano-Tech and Nano-Bionics(SINANO), Chinese Academy of Sciences.

1.2 Construction of experimental setup

The QCM system was put into a dark box with a laser source over the QCM chamber sealed with a transparent glass slide on the top. Under periodic UV irradiation of different wavelengths and power rates, a frequency shift signal in water was observed.

1.3 Photoresist evaluation

In our study, the positive photoresist AZ1518 was tested in the liquid state without a soft bake of photoresist to evaluate its typical photoresist properties. During experiment, a 500 μL of photoresist AZ1518 was injected into the QCM reaction chamber with a thickness of layer over 1mm, followed by exposure to 365nm ultraviolet light. The photochemical reactions of photoresists are monitored by QCM online via resonant frequency shift (ΔF) and energy dissipation signals.
